# Automated Sleep Stage Classification in Home Environments: An Evaluation of Seven Deep Neural Network Architectures

**DOI:** 10.3390/s23218942

**Published:** 2023-11-03

**Authors:** Jaime Yeckle, Vidya Manian

**Affiliations:** Department of Electrical and Computer Engineering, University of Puerto Rico, Mayaguez, PR 00681, USA; vidya.manian@upr.edu

**Keywords:** artificial neural networks, sleep stage classification, EEG signals

## Abstract

Sleep is an essential human physiological need that has garnered increasing scientific attention due to the burgeoning prevalence of sleep-related disorders and their impact on public health. Among contemporary challenges, the demand for authentic sleep monitoring outside the confines of specialized laboratories, ideally within the home environment, has arisen. Addressing this, we explore the development of pragmatic approaches that facilitate implementation within domestic settings. Such approaches necessitate the deployment of streamlined, computationally efficient automated classifiers. In pursuit of a sleep stage classifier tailored for home use, this study rigorously assessed seven conventional neural network architectures prominent in deep learning (LeNet, ResNet, VGG, MLP, LSTM-CNN, LSTM, BLSTM). Leveraging sleep recordings from a cohort of 20 subjects, we elucidate that LeNet, VGG, and ResNet exhibit superior performance compared to recent advancements reported in the literature. Furthermore, a comprehensive architectural analysis was conducted, illuminating the strengths and limitations of each in the context of home-based sleep monitoring. Our findings distinctly identify LeNet as the most-amenable architecture for this purpose, with LSTM and BLSTM demonstrating relatively lesser compatibility. Ultimately, this research substantiates the feasibility of automating sleep stage classification employing lightweight neural networks, thereby accommodating scenarios with constrained computational resources. This advancement aims at revolutionizing the field of sleep monitoring, making it more accessible and reliable for individuals in their homes.

## 1. Introduction

Sleep is a fundamental biological necessity that facilitates the restoration of vital physical and psychological functions, ultimately contributing to optimal performance. Scientific findings indicate that individuals dedicate approximately one-third of their lives to sleep, underscoring its pivotal role in maintaining overall health. However, the current fast-paced lifestyle has led to a surge in the prevalence of sleep-related disorders [1] among a growing number of individuals. Sleep disorders pose significant implications for neurological health, leading to compromised cognitive function, reduced performance levels, and diminished overall quality of life. Among the most-commonly encountered sleep disorders are sleep apnea, insomnia, and narcolepsy. Consequently, the investigation into sleep disorders has garnered substantial attention in recent years, recognizing its profound impact on individual well-being.

The first step in studying these disorders involves the classification of sleep stages. Traditionally, this research is conducted in external laboratories using various types of sensors, primarily to capture the electrical signals from the brain (EEG). In the laboratory, the subject sleeps while sensors record data, which are later stored and manually examined by a human expert, who is responsible for classifying the different stages of sleep.

The earlier techniques employed for sleep stage classification included traditional machine learning algorithms, which dominated the field and were extensively validated using publicly available datasets [2]. However, with the rapid expansion of neural networks and artificial intelligence, there has been a paradigm shift towards the adoption of Convolutional Neural Networks (CNNs) and Recurrent Neural Networks (RNNs) for this task, yielding promising outcomes. The integration of CNNs and RNNs has brought about significant improvements in addressing the challenges associated with manual sleep stage classification by expert evaluators. Notably, these sophisticated neural-network-based approaches have successfully mitigated the need for labor-intensive and time-consuming human evaluation, a prevailing concern in the domain. The utilization of neural networks, specifically CNNs and RNNs, has emerged as a cutting-edge approach in the field of sleep research, offering great potential for enhancing the efficiency and accuracy of automatic sleep stage classification. However, this approach demands large volumes of data and considerable computational resources, leading to extended model training times.

On the other hand, the recent development of portable sleep-monitoring devices has made it feasible to monitor sleep stages in home environments. However, this monitoring requires processing on devices with limited memory and processing capacity, which is not feasible with the complex structures of CNNs and RNNs, which have millions of parameters and high computational resource demands. Therefore, monitoring sleep stages in home environments with portable devices requires the development of new lightweight architectures for practical application.

To address the mentioned issues, lightweight models aimed at automatic sleep stage classification based on single-channel EEG signals have begun to emerge in scientific literature. Some of these models combine one-dimensional convolutional neural networks (1D-CNNs) with parameter reduction techniques. For example, Liao et al. [3] employed a 1D-CNN along with a Global Average Pooling (GAP) layer and an adaptive algorithm. Similarly, Zhou et al. [4] uses a 1D-CNN, GAP and a convolutional block to extract features, which were later fused with a residual block. In the study by Fiorillo et al. [5], a 1D-CNN and the Monte Carlo Dropout technique were used, while Zhou et al. [6] converted EEG data into spectrograms to accelerate the training process and enhance system performance.

Another group of research combines CNNs with RNNs, as performed by Zhu et al. [7] and Bresch et al. [8]. More recently, transformer techniques and attention mechanisms have demonstrated satisfactory results in natural language processing. Inspired by these advances, Yao et al. [9] implemented a convolutional network along with transformers, which were used to capture changing temporal features in the data. Attention mechanisms, combined with a CNN network, were also employed in the studies of Zhou et al. [10] and Yang et al. [11], demonstrating the feasibility of their integration into portable sleep-monitoring devices.

Overall, all these methods have significantly reduced the number of model parameters and the computational resources required. However, this has been at the expense of the algorithm’s ability to accurately detect different sleep stages, highlighting the ongoing need to develop new lightweight architectures for implementation in portable sleep-monitoring devices.

This research endeavors to tackle the challenge of identifying a simplistic neural network that can be efficiently implemented with limited computational resources. Our approach involves the evaluation of seven conventional neural network architectures, utilizing sleep data from a cohort of 20 subjects. Based on the results obtained, we meticulously analyzed the characteristics, advantages, and disadvantages of each architecture, serving as a pivotal basis for formulating a pioneering mobile-based methodology for home sleep monitoring. The primary objectives of this study were as follows: Firstly, we performed an automated classification of sleep stages employing traditional neural networks, and subsequently, juxtaposed the attained results with those reported in the existing literature. Secondly, we conducted an extensive analysis of the merits and limitations associated with the deployment of traditional models in sleep stage classification. Thirdly, we discerned the specific characteristics of traditional neural networks that engender enhanced performance in sleep classification within home environments.

Consequently, this evaluation revolves around the fundamental research inquiry: Can conventional neural network models effectively accomplish the classification of sleep stages? If affirmative, what specific characteristics of each model prove most apt for devising a novel lightweight architecture for sleep stage classification applicable within the subject’s home environment? This comprehensive knowledge base will serve as the foundation for proposing innovative research directions aimed at enhancing the performance of automatic sleep stage classification, in particular utilizing wearable EEG technology in real-world settings. By addressing these aspects, the study endeavors to significantly contribute to the advancement of sleep research and foster improvements in sleep stage classification methodologies, enabling practical and reliable monitoring solutions for home-based sleep analysis.

The remainder of this paper is structured as follows: Section 1 provides the Introduction, delineating the objectives and significance of this study. Section 2 expounds upon the methodology employed. Section 3 delineates the outcomes derived from the evaluation of various neural networks for the classification of sleep stages. Section 4 engages in a comprehensive discussion of the findings, and lastly, Section 5 encapsulates the conclusions drawn from this research.

## 2. Methods

**Figure 1:**
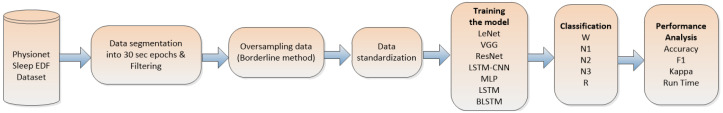
Methodology workflow.

This section outlines the methodology employed to assess the efficacy of state-of-the-art neural-network-based models for sleep stage detection, utilizing a single-channel EEG signal. Each EEG epoch corresponds to a sleep stage, and these epochs are subjected to evaluation by various neural networks. The selection of these networks was based on their status as the state-of-the-art in time series recognition [12], thereby encompassing a diverse array of models with unique attributes. This approach enables a comprehensive comparison of each model’s effectiveness, measured through key metrics such as the accuracy, kappa, F-value, and time. By employing these evaluation criteria, we aimed to gain valuable insights into the performance and suitability of each neural network model for sleep stage classification based on single-channel EEG data. Figure 1 shows a workflow of the methodology.

### 2.1. Dataset

In this study, we utilized the publicly available Physionet’s Sleep-EDF databank [13]. This dataset comprises recordings from 20 subjects, each containing polysomnographic data. The recordings consist of a single EEG channel acquired from the Fpz–Cz electrode location and are saved in corresponding EDF files. The sleep data consist of time series, where the horizontal axis represents the time variable and the vertical axis corresponds to the millivolts captured by the electrode of the Fpz channel. Notably, each subject has two nights of recorded data, except for Subject 13, where data loss occurred due to initial storage on tapes. The EEG channel signals were sampled at a frequency of 100 Hz, resulting in 30 s EEG epochs, each containing 3000 data points. These epochs were meticulously classified into one of five sleep stages based on the American Academy of Sleep Medicine (AASM) rules by well-trained human experts. The five sleep stages include Wakefulness (W), Stage 1 (N1), Stage 2 (N2), Stage 3 (N3), and Rapid Eye Movement (REM). For our work, we performed five-state classification involving the identification of wakefulness, Stage 1, Stage 2, Stage 3, and REM sleep, as summarized in Table 1, which outlines the key characteristics of the dataset utilized in our study.

#### Balance of Classes in Dataset

The sleep dataset for the 20 patients encompasses five distinct classes corresponding to different sleep states, namely, Wakefulness (W), Stage 1 (N1), Stage 2 (N2), Stage 3 (N3), and Rapid eye movement (R). However, it is important to note that the distribution of samples across these classes is imbalanced, leading to varying numbers of instances in each class. Consequently, there exists a majority class with a higher number of samples and a minority class with fewer samples. When evaluating neural network models using imbalanced data, the traditional accuracy metric tends to favor better predictions for the majority class at the expense of the minority class. As a result, the accuracy metric may not accurately reflect the model’s predictive performance for the minority class, and its reliability in such scenarios is limited. Hence, alternative evaluation metrics that consider the class imbalances are essential for a comprehensive assessment of the model’s effectiveness.

To address the class imbalance, we implemented two distinct approaches: algorithm-level and data-level improvements. At the algorithm level, we devised modified versions that incorporated an additional cost for misclassifying instances from the minority class during the training process. By imposing penalties, the algorithm is encouraged to pay greater attention to the minority class, thereby enhancing its performance in handling imbalanced data. In our investigation, we harnessed the “class_weight” option available in the Keras library, which we utilized to implement our neural network models.

At the data level, we adopted a strategy involving the alteration of the imbalanced data distribution prior to commencing the algorithm training. In our study, we adopted the Borderline method, proposed by Han et al. [14]. This method is founded on the concept that samples located at the boundaries of each class are more susceptible to misclassification compared to those situated further away from the boundary. As such, these boundary samples hold greater significance for the classification process. Building upon this premise, the Borderline method employs an oversampling technique for the minority sleep state samples situated at the class boundary, generating synthetic samples to balance the class distribution. It is important to highlight that Borderline is derived from the Synthetic Minority Over-sampling Technique (SMOTE) method, but with the key distinction that it exclusively oversamples the minority samples located at the class boundary, thereby enhancing the handling of class imbalance in the dataset.

**Figure 2:**
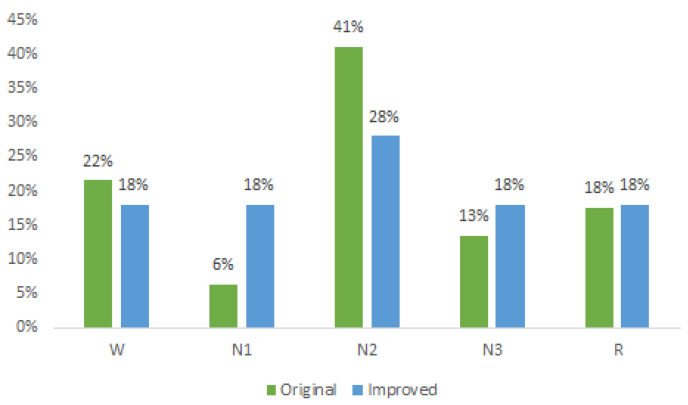
Original and improved dataset.

In Figure 2, the original distribution of each sleep state is depicted in green, with the N2 state accounting for the highest percentage (41%), while the N1 state holds the smallest proportion (6%) of samples. The blue color represents the distribution after applying the Borderline method, resulting in improved class proportions. Notably, the states W, N1, N3, and R each exhibited an approximate 18% representation, while the N2 state comprised 28% of the dataset.

This revised distribution achieves a more-balanced representation compared to the original dataset. Ideally, equal representation for all classes at 20% would be optimal; however, such an equal distribution would significantly augment the dataset and, consequently, increase the processing time. Nonetheless, with the attained balancing, significant improvements in the classification results were observed, surpassing those reported in the existing literature, as elaborated in the subsequent section.

### 2.2. Evaluated Network Models

Artificial Neural Networks (ANNs) represent a fundamental milestone in the field of artificial intelligence and have revolutionized various domains, including pattern recognition, natural language processing, image and speech recognition, and data analysis. ANNs are computational models that mimic the functioning of the human brain, enabling machines to learn, generalize, and make predictions. ANNs consist of interconnected artificial neurons, or nodes, organized into layers, and these networks can learn from data and adapt their connections to improve their performance. The most-important types of ANN are the Convolutional Neural Networks (CNNs) and Recursive Neural Networks (RNNs), and they have significantly advanced the field of deep learning by enabling the analysis and understanding of complex data patterns. A convolutional neural network is a feed-forward network with many layers, the output of one layer being the input to the next. The information flows uni-directionally from the input, then passes to the hidden layers and, finally, to the output. For an input signal of one-dimensional $x_{n}$ with N samples, the CNN can be defined as:

(1) $$y_{n}=\sum_{i=0}^{M-1}h_{i}x_{n-i}$$

where *h* is a filter of one dimension of size *M*. For CNNs with a higher dimension, the number of summations increases in relation to the dimensions. Likewise, a CNN usually comprises convolution, pooling, and connected layers. The convolution layer is used to extract features by learning from the input signal; in other words, the input sample is convolved with a kernel, and then, the output is used in the subsequent layer. The pooling layer merges semantically similar features into one (i.e., reducing the spatial dimension) and usually maximizes the operation, while a fully connected layer serves as a classifier, connecting neurons with all subsequent neurons in the next layer. CNNs have revolutionized computer vision applications. They are instrumental in image classification tasks, where they can classify objects and scenes with high accuracy.

On the other hand, RNNs find extensive use in natural-language-processing tasks. RNNs employ a recursive approach, i.e., they use feedback loops and process an input sequence one element at a time, maintaining in their hidden units a state vector that implicitly contains information about the history of all the past elements of the sequence. The most-basic RNN is given by:

(2a) $$\begin{array}{cc} y_{t} & =\varphi (W_{hy}h_{t}) \end{array}$$

(2b) $$\begin{array}{cc} h_{t} & =\varphi (W_{hh}h_{t-1}+W_{xh}x_{t}) \end{array}$$

where $h_{t}$ represents the state of the system at time *t* with input $x_{t}$, φ(·) is an activation function, and $W_{hy},W_{hh},W_{xh}$ are weight matrices. The most-common type of RNN is the Long Short-Term Memory (LSTM). It incorporates a memory to mitigate the gradient problem, which arises because the parameters are shared across time and cause the gradient to become smaller or larger as the data move through each time step.

The integration of CNNs and RNNs has yielded significant advancements across various domains. For instance, in image captioning, CNNs are employed to extract visual features, which are subsequently fed into RNNs to generate descriptive captions. Likewise, in video analysis, CNNs extract features from each frame, and RNNs capture the temporal dependencies between these features, facilitating a comprehensive understanding of the video context.

**Figure 3:**
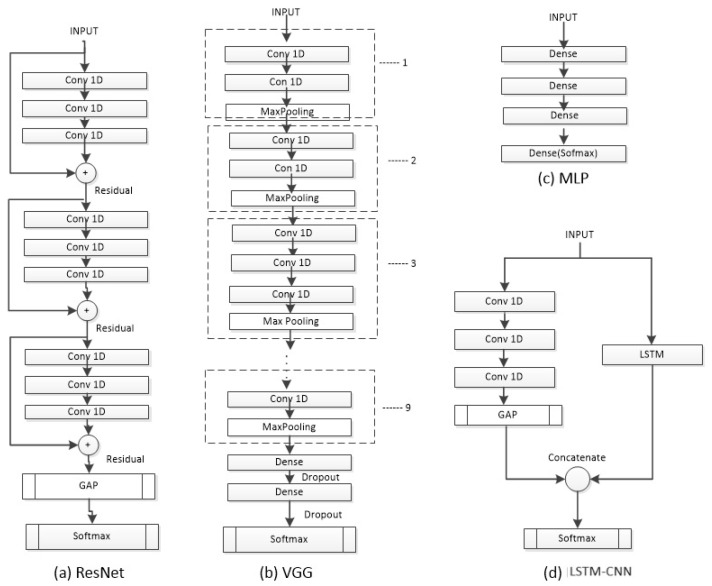
The architectures ResNet, VGG, and LSTM-CNN used to evaluate the dataset.

**Figure 4:**
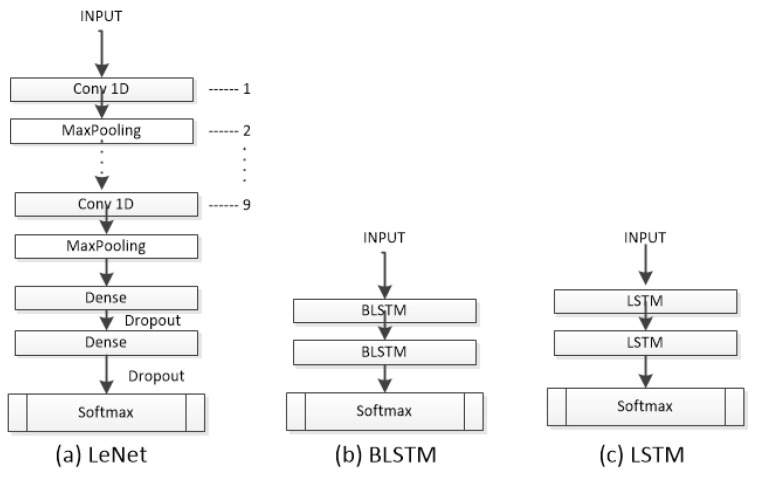
The architectures LeNet, BLSTM, and LSTM used to evaluate the dataset.

To evaluate the classification of sleep stages, we employed state-of-the-art neural-network-based models (see Figure 3 and Figure 4). These models were selected based on their proficiency in time series recognition and their representation of diverse attributes. Each architecture was trained with its recommended optimizer, learning rate, and batch size as suggested in its respective literature. In general, the implementation of the methods was based the following methodology: First, the parameters recommended by the literature for each model were tested. Then, adjustments were made with the strategy of using a subset of data and making improvements until an optimal result was achieved. Finally, the models were tested with the entire dataset.

The details of the parameters and the training scheme are described below for each evaluated architecture.

#### 2.2.1. Multi-Layer Perceptron

The utilized version corresponded to Wang’s [15], comprising three Fully Connected (FC) layers, each equipped with 500 neurons and employing Rectified Linear Unit (ReLU) as the activation function. The final layer is a Softmax layer, containing a number of neurons equivalent to the classes present in the data under evaluation. In order to mitigate the risk of overfitting, the neural network incorporates the dropout technique after each layer, applying distinct dropout percentages. During the training process, the network was optimized using the Adadelta optimizer, with a learning rate of 0.1 and a batch size of 256. These choices aimed to enhance the neural network’s performance and ensure appropriate model generalization during the classification task.

#### 2.2.2. Long Short-Term Memory Fully Convolutional Network

The model is a hybrid of LSTM and a CNN referred to as an LSTM-CNN [16]. An LSTM-CNN is a two-stream network that combines a fully convolutional stream and a recurrent stream. The convolutional stream has three layers with 128, 256, and 128 filters, respectively, incorporating batch normalization to prevent overfitting. The LSTM stage consists of 128 neurons and uses a dropout with a rate of 0.8. The input is separately fed into the CNN and LSTM stages, and then, both streams are concatenated before entering the final Softmax layer (5 neurons). The neural network was trained using the Adam optimizer, a learning rate of 0.001, and a batch size of 32.

#### 2.2.3. Residual Network

A Residual Network (ResNet) [17] is a deep CNN that uses residual connections between blocks. The network has three residual blocks with varying filter lengths and pooling. Each residual block contains three convolutional layers. The residual connection connects the input of each residual block to the input of the next block using an addition operation. The last two layers of the ResNet include a Global Average Pooling (GAP) layer (calculates the average output of each feature map) and an output layer with Softmax (converts a vector of numbers into a vector of probabilities proportional to the original vector). The ResNet was trained using the Adam optimizer, a learning rate of 0.001, and a batch size of 128.

#### 2.2.4. Visual Geometry Group Network

The Visual Geometry Group (VGG) model is a CNN [18], which employs 1D-CNN and adapts to the input length of the network. The VGG consists of nine stages, where each stage comprises two or three CNN layers with varying numbers of filters (64, 128, 256, 512). For the classification part, it utilizes two Fully Connected (FC) layers with an equal number of neurons (4096), ReLU as the activation function, and dropout after each layer to prevent overfitting with a rate of 0.5. The last layer is a Softmax layer with five neurons (equal to the number of classes). The neural network was trained using the SGD optimizer, a learning rate of 0.001, and a batch size of 64, as suggested in the original VGG’s hyperparameters.

#### 2.2.5. Lecun Network

The Lecun Network (LeNet) [19] is a CNN with basic units such as a convolutional layer, pooling layer, and fully connected layer. The LeNet works with the data by dividing it into smaller sets through convolutions; it then reduces the parameters within a pooling layer, which filters the data by finding key features. The LeNet is composed of nine 1D-CNN layers with different numbers of filters (16, 26, 36, 46, 56, 64, 66, 76, 86). The classification part consists of two FC layers with 120 and 84 neurons, respectively, using the ReLU activation function. Like most neural models, it incorporates a dropout layer after each FC layer to prevent overfitting and a final Softmax layer with five neurons. The LeNet was trained using the Adam optimizer, a learning rate of 0.01, and a batch size of 128.

#### 2.2.6. Long Short-Term Memory

LSTM [20] is a popular variant of RNNs. LSTM allows you to select which information should be remembered over time and which should be forgotten. LSTM cell networks are mainly used for text recognition, and in this work, we utilized a module with 512 neurons and a Softmax layer with 5 neurons as the output. The LSTM was trained using the Adam optimizer, a learning rate of 0.001, and a batch size of 32.

#### 2.2.7. Bidirectional Long Short-Term Memory)

Bidirectional LSTMs (BLSTMs) [21] are an adaptation of LSTMs that employ both a forward and a backward recurrent connection. Concatenation is also used by the BLSTM for output merging. It is intended to compare the effects of having forward and backward recurrent connections on sleep data using both a regular LSTM and a bidirectional one. BLSTM is built on LSTM, and this model was trained with the same configuration as the LSTM.

### 2.3. Evaluation

In accordance with the information presented in Figure 1, the primary stage of the methodology consisted of dividing the data into consecutive 30 s epochs. Subsequent to this step, we proceeded to eliminate epochs containing noise and those lacking proper sleep stage labels from the dataset. To address class imbalance, we applied the Borderline method, which ensured an equitable distribution of sleep states. After completing the aforementioned steps, the data underwent standardization. Following this, the classifier was subjected to training and testing utilizing the extracted features, and subsequently, a comprehensive evaluation of the entire process was performed.

To obtain our results, a 20-fold cross-validation approach was employed to assess the generalizability of our strategy. Within each fold, one subject’s recording was exclusively used for testing, while the remaining subjects’ recordings were utilized for training. Notably, each subject was tested only once, ensuring a one-to-one correspondence between the cross-validation folds and test subjects.

During training and testing, the models adhered to their respective literature’s recommended hyperparameters, including the optimizers, learning rates, and batch sizes. The number of iterations was kept constant across the dataset, with no early stopping applied, to provide each model with equal opportunities. Performance metrics such as the accuracy, kappa, F-value, and runtime were obtained from these tests, and subsequently, the average of the accuracies was calculated once all 20 folds had concluded.

All experiments were conducted on an MSI Aegis R Desktop with a 13th-Gen Intel Core i7 processor (16-Core), 32 GB DDR4 main memory, GeForce RTX 3060 12GB GDDR4 Graphics Card, 1TB Solid State Drive, 2TB Hard Drive, and Windows 11 64 bit operating system. The programming language used was Python 3.9. Additionally, the Keras and TensorFlow 2.10 libraries were employed for the implementation of the deep learning models, all within a Miniconda development environment. These specifications ensured a reliable and consistent testing environment for the evaluation of the models’ performance.

## 3. Results

**Figure 5:**
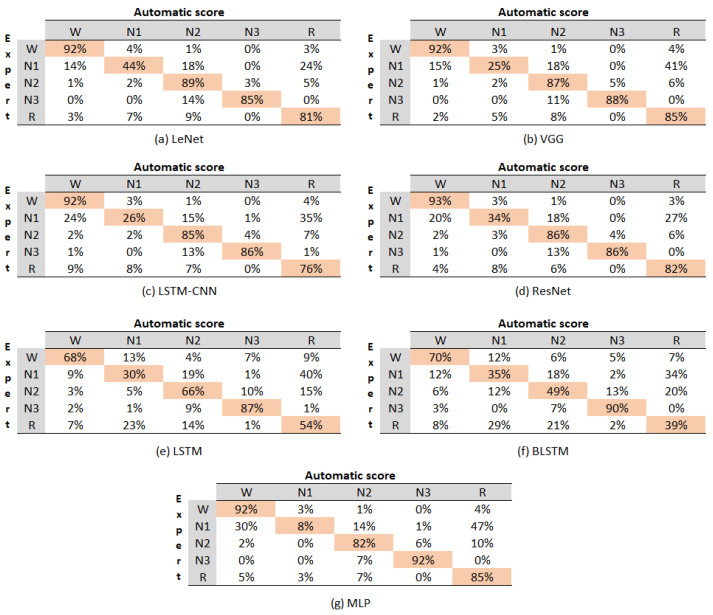
Confusion matrix of the evaluated models.

Figure 5 displays the confusion matrix for all the evaluated models, with each matrix representing the aggregation of the confusion matrices from each cross-validation fold.

The values within the matrices indicate the percentage of instances that the algorithm classified as belonging to the corresponding class denoted by the column. For instance, in the case of the LeNet model, the value in Row 1, Column 1 signifies that, out of 100 instances of class W, the model accurately predicted 92%. It was evident that the LSTM and BLSTM models yielded the lowest performance results. Therefore, they were not included in the analysis of the classification accuracy for each state. The sleep stage with the highest accuracy across most models was W, achieving an average accuracy of 92%. The subsequent stages in terms of accuracy were N3 (88%), followed by N2(86%), R (82%), and N1 (27%). Notably, it is essential to mention that the N1 stage consistently exhibited confusion with the R stage across all the confusion matrices.

Upon the overall evaluation, the models can be ranked from best to worst in terms of accuracy for sleep stages as follows: LeNet, VGG, ResNet, MLP, LSTM-CNN, LSTM, and BLSTM. This ranking highlights the models’ varying performance levels in classifying different sleep stages, with LeNet showing the highest accuracy and BLSTM demonstrating the lowest accuracy among the evaluated models.

All the training graphs exhibit a similar behavior. Among the most-notable characteristics, we observed that the training accuracy curve consistently surpassed the validation set curve. This phenomenon arose due to the implementation’s utilization of 30% of the total 19 subjects as the validation set, while the test set consisted of a single subject. As depicted, it was relatively easier to predict the sleep stage of a single patient compared to the challenge of predicting the sleep stages of multiple subjects.

Furthermore, all models demonstrated a training curve that started above 50% accuracy in the initial epoch. This initial surge in accuracy was attributed to the CNN’s capacity to learn data characteristics effectively. However, the MLP model exhibited a different behavior due to its nature of having fully connected layers.

**Figure 6:**
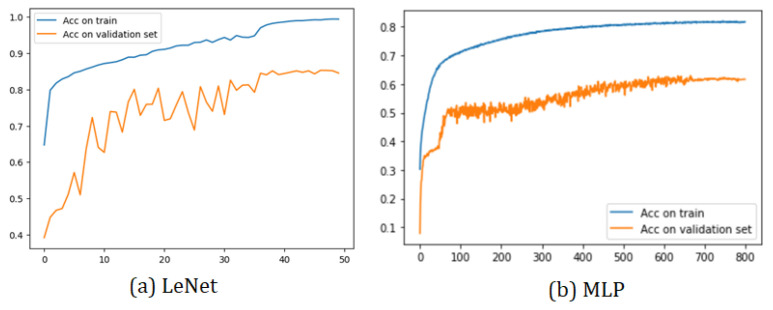
Training plots of LeNet and MLP.

Two sample training graphs (LeNet and MLP) showcasing the aforementioned characteristics are presented in Figure 6. These training graphs provide valuable insights into the models’ performance and generalization capabilities during the training process. They highlight the impact of the dataset size and composition on the model accuracy during validation, which is essential for understanding the models’ effectiveness in real-world applications.

Table 2 presents the obtained metrics, showcasing the performance of the evaluated models. Again, the LSTM and BLSTM models exhibited the poorest results; thus, they were excluded from subsequent analysis. The remaining models achieved accuracy levels ranging from 85% to 82%, with LeNet emerging as the top-performing model (85%). This outcome indicated that all models performed well. Moreover, considering the inherent variability in inter-rater agreement among human sleep evaluators, it is crucial for the models not to overfit to the scoring style of specific human evaluators, as mentioned in [22]. In our datasets, each sleep record was rated by one of six different evaluators, with 27 records annotated by a single evaluator and the remaining 12 records rated by the other five evaluators. The kappa coefficient results ranged from 79% to 75%, signifying substantial agreement between the evaluated models and a classifier that merely guesses at random, based on the frequency of each sleep stage class.

Examining the F-score, we observed values in the range of 85% to 81%, demonstrating high weighted class precision, consistent with the overall accuracy achieved. Among the evaluated metrics, namely the accuracy, kappa, and F-score, the best results were attained by the LeNet, VGG, ResNet, MLP, and LSTM-CNN models, ranked from best to worst. However, when considering the runtime metric, the order shifted. LeNet exhibited the shortest execution time with 2 min, followed by MLP, which took 17 min, LSTM-CNN taking 22 min, VGG taking 30 min, and ResNet taking 65 min. Consequently, LeNet emerged as an excellent model for classifying sleep data, demonstrating strong performance in terms of accuracy and algorithmic runtime.

In summary, the comprehensive evaluation demonstrated an overall improvement in all performance metrics. Notably, the LeNet, VGG, and ResNet models exhibited superior results compared to the other models, achieving an accuracy of 85%, 84%, and 84% respectively. However, it is crucial to highlight that not all evaluated architectures had the same processing time.

### Performance Comparison

The performance of the analyzed architectures was compared with recent studies that used the same Sleep-EDF databank [13], i.e., twenty healthy subjects using the channel Fpz–Cz. Table 3 shows the results obtained, together with the model architectures, and their accuracy and kappa parameters for comparison. The LeNet, ResNet, and VGG models outperformed the models found in the literature. It is worth highlighting that, in addition to its strong performance, LeNethad a very short processing time. This makes LeNet a promising candidate to be considered for sleep monitoring in patients’ home environments. For the runtime metric, we did not find previous studies that allowed us to make a comparison. However, in our experiment, we observed that the runtimes were high: an average of 1 h for the ResNet model and 30 min on average for LSTM and BLSTM. We must mention that high computational resources were required to achieve these results, as detailed in the Method Section. In conclusion, given the results obtained and the resources used, implementing these methods in portable systems (outside a sleep study laboratory) presents new challenges, which we detail in the next section.

## 4. Discussion

### Analysis of Advantages and Disadvantages of Models Evaluated

The sleep dataset utilized in the present research comprises time series data, systematically sampled at a frequency of 100 samples per second. The data were segmented into discrete epochs, with each epoch encompassing a temporal span of 30 s. Subsequently, the data were subjected to analysis using various computational models, thereby yielding diverse outcomes for each model, as summarized and presented in Table 2.

The LSTM and BLSTM models demonstrated inadequate performance, achieving results below the acceptable 70% threshold across all quantitative evaluation metrics. In their present form, these architectural configurations are deemed unsuitable for accurately classifying sleep stages. The suboptimal performance can be attributed to the feedback structure intrinsic to these neural networks, which proves problematic when applied to lengthy input sequences of sleep data (in our case, 3000 time steps). Additionally, the nature of sleep data being tabular renders them unsuitable for recurrent neural network models (LSTM, BLSTM), further contributing to the diminished performance of LSTM and BLSTM in this context.

The four most-favorable outcomes were achieved by LeNet, VGG, and ResNet respectively. Notably, the three models are classified as one-dimensional Convolutional Neural Networks (1D-CNNs), although they exhibited differences in terms of the number of employed CNN layers and the arrangement of the pooling layers.

The LeNet architecture demonstrated superior performance, exhibiting outstanding results across all performance metrics, with an impressive accuracy of 85%. This model prominently comprises nine convolutional layers, and its salient characteristic were not shared by other models under consideration with an immediate application of pooling operations after each convolutional layer. This strategic design choice significantly reduces the data dimensions and expedites the processing time. Moreover, in the LeNet model, the number of convolutional layers is dynamically adapted relative to the size of the input data sequence, and the convolutional filters are distinct in number for each convolution. These distinctive architectural elements synergistically contribute to the model’s exceptional capacity in efficiently discerning and classifying sleep stages within each epoch.

The VGG architecture attained an accuracy of 84%, notable for its adoption of a deep stack of 25 convolutional layers organized into nine distinct phases. Within each phase, the convolutional layers conduct 2 or 3 convolutions, employing a wide range of distinct filters, culminating in a maximum of 512 filters. Subsequently, the pooling operations are performed at the termination of each phase. Nevertheless, the VGG architecture exhibited a discernible limitation in the form of a lengthier processing time when compared to the LeNet model.

The ResNet architecture demonstrated a classification accuracy of 84% through the utilization of nine convolutional layers distributed across three successive phases. An essential feature of ResNet lies in its adoption of residual connections, which involve the summation of input data flow from a given phase with the output of the same phase. This ingenious mechanism enables seamless information flow through the network, facilitating gradient propagation and alleviating vanishing/exploding gradient issues. Despite its promising performance, the ResNet model is subject to a noteworthy limitation, manifesting in its extended processing time, requiring approximately 65 min for completion.

The Multi-Layer Perceptron (MLP) architecture exhibited a remarkable accuracy of 82%, operating without convolutional layers in its design. This unique model relies exclusively on three fully connected layers for its predictive capacity. However, it is crucial to highlight that the MLP architecture necessitates a substantial number of iterations, amounting to approximately 800, which surpassed the iteration count observed in preceding neural network architectures, where the number of iterations typically remained below 50.

Conversely, the LSTM-CNN hybrid architecture, integrating three convolutional layers in parallel with one LSTM module, achieved sleep stage classification with an accuracy of 82%. Remarkably, this hybrid model yielded results comparable to those achieved by previous neural network architectures, while concurrently maintaining a reasonable processing time of 22 min.

The findings suggest that convolutional layers serve as integral components in the acquisition and learning of the intrinsic characteristics of various sleep stages. Additionally, the integration of convolutional layers in conjunction with immediate pooling methodologies yielded notable enhancements in accuracy and computational efficiency when analyzing sleep stages. Furthermore, the employment of fully connected layers demonstrated its utility in facilitating the accurate identification and classification of sleep stages, as effectively demonstrated by the LeNet and MLP models.

## 5. Conclusions

In the present study, we addressed the challenge of identifying a streamlined neural network architecture for the classification of sleep stages, designed for efficient implementation on resource-constrained computational platforms. Initially, we implemented seven established deep learning architectures, widely recognized in the field. Our comprehensive analysis of the results revealed that three of these architectures—specifically, LeNet, VGG, and ResNet—outperformed recent state-of-the-art methodologies reported in the literature. Subsequently, we conducted an in-depth evaluation of each of the instantiated architectures to elucidate their individual strengths and limitations in the context of home-based sleep monitoring. Our observations indicated that LeNet emerged as the most-favorable choice for deployment within a home environment, while LSTM and BLSTM exhibited less suitability. In conclusion, this research not only underscores the feasibility of automated sleep stage classification within a home setting, but also underscores the potential of lightweight neural networks to enable such automation, even in scenarios with limited computational resources.

## Figures and Tables

**Table 1:** Sleep dataset description.

Description	Value
Epoch size	30 s
Channel	Fpz–Cz
Sampling rate	100
No. of subjects	20
No. sleep stages	5
No. of recorded days	2

**Table 2:** Accuracy, F-score, runtime, and kappa coefficient for all models.

Metric	LeNet	ResNet	VGG	MLP	LSTM-CNN	LSTM	BLSTM
Accuracy	0.85	0.84	0.84	0.82	0.82	0.65	0.56
Kappa	0.79	0.77	0.78	0.75	0.75	0.55	0.43
F	0.85	0.84	0.84	0.81	0.81	0.66	0.57
Run time (min)	2	65	30	17	22	32	27

**Table 3:** Comparison with other methods using Sleep-EDF dataset (subjects = 20, channel = Fpz–Cz).

Architecture	Accuracy	Kappa
Multitask CNN [23]	0.82	0.75
DeepSleepNet [24]	0.82	0.76
Scattering spectrum diffusion map [25]	0.82	0.76
Stacked sparse autoencoders [26]	0.79	Not Given
LeNet ^1^	0.85	0.79
ResNet ^1^	0.84	0.77
VGG ^1^	0.84	0.78
MLP ^1^	0.82	0.75
LSTM-CNN ^1^	0.82	0.75

^1^ Architectures evaluated in this paper.

## Data Availability

Publicly available datasets were analyzed in this study. This data can be found here: https://physionet.org/content/sleep-edfx/1.0.0/.

## Abbreviations

The following abbreviations are used in this manuscript:

CNN	Convolutional Neural Networks
RNN	Recurrent Neural Network
LSTM	Long Short-Term Memory
